# The Effect of Zinc and Selenium Supplementation Mode on Their Bioavailability in the Rat Prostate. Should Administration Be Joint or Separate?

**DOI:** 10.3390/nu8100601

**Published:** 2016-10-04

**Authors:** Adam Daragó, Andrzej Sapota, Marzenna Nasiadek, Michał Klimczak, Anna Kilanowicz

**Affiliations:** Department of Toxicology, Faculty of Pharmacy, Medical University of Lodz, Muszynskiego 1, 90-151 Lodz, Poland; andrzej.sapota@umed.lodz.pl (A.S.); marzenna.nasiadek@umed.lodz.pl (M.N.); michal.klimczak@umed.lodz.pl (M.K.); anna.kilanowicz@umed.lodz.pl (A.K.)

**Keywords:** zinc gluconate, sodium selenite, selenomethionine, supplementation, rats, prostate, metallothionein-like proteins

## Abstract

It is thought that zinc and selenium deficiency may play a significant role in the etiology of prostate cancer. Although joint zinc and selenium supplementation is frequently applied in the prevention of prostate diseases, the bioavailability of these elements in the prostate after co-administration is still unknown. The study examines the effect of subchronic supplementation of zinc gluconate and selenium compounds (sodium selenite or selenomethionine), administered together or separately, on their bioavailability in the prostate, as well as the induction of metallothionein-like proteins (MTs) bound to zinc in the prostate and liver. Zinc concentration in the dorso-lateral lobe of the prostate was significantly elevated already after the first month of supplementation of zinc alone. In the supplementation period, the MTs level increased together with zinc concentration. In contrast, the ventral lobe of the prostate did not demonstrate significantly higher levels of zinc until after three months of supplementation, despite the MTs induction noted after one-month supplementation. Increased selenium levels in the dorsolateral lobe were observed throughout the administration and post-administration periods, regardless of the selenium compound used or whether zinc was co-administered. The results of our studies suggested for the first time that these elements should not be administered jointly in supplementation.

## 1. Introduction

Recent years have seen growing interest in research studies investigating the role of zinc and selenium supplementation, especially in the prevention of neoplastic diseases including prostate cancer (PCa). PCa continues to be one of the most common fatal cancers in men [1], and aging is believed to be a major risk factor. Prostate cancer is rare in men younger than 40, but the chance of developing PCa rises rapidly after age 50. About 60% of prostate cancer cases are found in men older than 65 [2]. The occurrence of PCa is not only believed to be closely associated with age, but also with oxidative stress and disturbed zinc homeostasis in the prostate. Studies show that excessively low zinc concentrations in the cells of the prostate can also play a significant role in the development of PCa [3,4,5,6]. A low zinc concentration in the prostatic gland may be responsible for intensified citrate oxidation and changed ATP concentration, which can ultimately induce carcinogenic processes or lead to tumor growth [7,8].

Numerous studies report the presence of significant zinc deficiencies in the elderly population of Europe, regardless of the country of residence [9,10,11]. Optimal daily level of zinc supplementation ranges from 30 to 85 mg [12,13,14]. Although it is not known whether dietary zinc intake affects intraprostatic zinc levels, our earlier studies on rats have indicated that oral administration of zinc significantly increases its concentration in the prostate, but its availability in this organ varies based on individual zinc preparations, which may be based on differences in absorption associated with zinc preparations [15]. Wegmüller et al. (2014) notes that in humans, zinc gluconate, like citrate, should be recommended for the prevention of zinc deficiency [16].

Selenium is another element used in the prevention of cancer (e.g., PCa). It has been shown that selenium exhibits antineoplastic properties by inhibiting the development of reactive oxygen species (ROS), thus protecting cells against oxidative DNA damage [17]. The recommended mean selenium intake is 60 μg/day for men and 53 μg/day for women [18]. The Nutritional Prevention of Cancer trial showed selenium-enriched yeast supplements (200 μg/day) had a protective effect on total cancer mortality, and also on total cancer incidence [19]. However, it should be taken into account that an adult should not consume more than 400 μg selenium/day [20,21].

A literature review reveals that despite numerous experimental animal studies, carried out to investigate the influence of both elements on different organs when administered separately, nothing is known about their effects on the prostate (including bioavailability), especially when administrated jointly. Similarly, no data exist concerning the effect of long-term use of zinc + selenium supplementation on the bioaccumulation of both elements in the prostate; an important point considering that interactions may occur between these two elements, as demonstrated by Maret (2000), who reports that joint selenium and zinc administration may induce significant interactions in the metallothionein/thionein system which may not always be beneficial [22].

The aim of the present study is to assess the effect of subchronic (90-day) supplementation of male rats with zinc gluconate and selected selenium compounds, selenomethionine (SeMet) and sodium selenite (Se), administered orally by gavage (jointly or separately) on their bioavailability in the prostatic tissues. Of particular interest are the effects on the ventral lobe, where zinc levels are known to decrease with age, and on the induction of metallothionein-like proteins (MTs) bound to zinc in the prostate and liver.

## 2. Materials and Methods

### 2.1. Animals

One hundred and eighty male Wistar rats (16 weeks old, weighing 290–320 g) from the breeding colony of the Medical University of Lodz, were subjected to a two-week acclimatization period. The animals were maintained in clean polypropylene cages under controlled conditions (23 ± 1 °C, 12-h light/dark cycle, relative humidity of 50% ± 10%) and were fed a standard pelletized “Murigran” diet, with tap water supplied ad libitum. Mean zinc and selenium levels in feed were determined. Feed and water intake were regularly checked throughout the entire experiment. The experiments were performed with the permission of the Local Ethical Committee for Experimentation on Animals of Lodz, Poland (Resolution No. 43/LB479/2009).

The study was conducted over a period of 270 days: A 90-day administration period and a 180-day post-administration period. Each of the 25 experimental groups and each of the five control groups comprised six males. All the rats were weighed daily immediately prior to administration. The animals were given the aqueous solution of 0.5 mL/100 g body weight (b.w.) zinc and/or selenium compounds by gavage at the following daily doses:

- Zn: Zinc gluconate (C_12_H_22_O_14_Zn·xH_2_O), 5.0 mgZn/kg b.w.
- Se: Sodium selenite (Na_2_SeO_3_), 2.8 μgSe/kg b.w.
- SeMet: Selenomethionine (CH_3_SeCH_2_CH_2_CH(NH_2_)COOH), 2.8 μgSe/kg b.w.
- Zn + Se: Zinc gluconate, 5.0 mgZn/kg b.w. and sodium selenite, 2.8 μgSe/kg b.w.
- Zn + SeMet: Zinc gluconate, 5.0 mgZn/kg b.w. and selenomethionine, 2.8 μgSe/kg b.w.

The control groups received water in an identical manner as zinc and/or selenium compounds.

The zinc compound used for supplementation was selected on the basis of the outcome of an earlier study [15], in which zinc gluconate was found to exhibit the highest bioavailability in the rat prostate. After conversion, the applied zinc and selenium doses corresponded to the average level of recommended dose for supplementation in humans. Zinc gluconate (puriss grade) was purchased from Alfa Aesar GmbH & Co KG (Karlsruhe, Germany). Sodium selenite and selenomethionine (puriss grade) were purchased from Sigma-Aldrich CHEMIE GmbH (Steinheim, Germany).

After a 90-day administration period, the animals were observed for the following 180 days. In the post-administration period, the animals of the experimental and control groups had free access to standard feed and water.

On days 31, 61 and 91 of the administration period and on days 91 and 181 of the post-administration period, six animals from the experimental groups and control group were sacrificed by bleeding under light CO_2_ narcosis. The blood was collected into Sarsted tubes for metal analysis, and, within 4 h of sampling, part of the blood was centrifuged at 1620× *g* for 10 min in a refrigerated centrifuge at 4 °C to separate the plasma. The buffy coat was removed, the plasma separated and the remaining erythrocytes drawn from the bottom. They were washed three times in cold saline (9.0 g/L NaCl), and hemolyzed by the addition of an equal volume of ice-cold demineralized ultrapure water (MilliQ plus reagent grade; Millipore) to yield a 50% hemolysate. The plasma and erythrocyte hemolysate were stored at −80 °C in cryo-tubes for biochemical assay.

The liver and prostate tissues were removed from the adhering connective tissue and accurately weighed. The prostate was divided into dorso-lateral (DL) and ventral (V) lobes as described previously [15]. The tissues were stored at −80 °C in cryo-tubes for further analyses.

### 2.2. Element Determinations

Zinc and copper were determined in the blood, liver and prostate after mineralization by flame atomic absorption spectrometry (Avanta PM, GBC Scientific Equipment). The limits of detection, calculated as concentrations corresponding with an absorption value equal to a three-fold standard deviation of the signal for the lowest standard, were 0.034 μg/mL for zinc and 0.018 μg/mL for copper. A calibration curve was obtained using a range of concentrations of a solution of the two metals (ASTASOL, Prague, Czech Republic). The graph obtained was linear in the concentration range described below, and the equations of the curve were as follows:

Zn (0.05 − 1.5 μg/mL) *y* = 0.2049*x* + 0.00620; *R^2^* = 0.9971

Cu (0.05 − 0.5 μg/mL) *y* = 0.1033*x* + 0.00012; *R^2^* = 0.9998

Selenium determinations in blood, liver and prostate were performed on a Hitachi F-4500 spectrofluorometer according to Danch and Drozdz (1996) [23]. The limit of detection was 0.012 μg/mL. The graph obtained was linear in the concentration range described below, and the equation of the curve was as follows:

Se (0.05 − 1.6 μg/mL) *y* = 225.77*x* + 8.4174; *R^2^* = 0.9982

Concentrations of zinc, copper and selenium in investigated tissues were expressed as μg/g wet tissue or μg/mL blood.

The intra-laboratory quality control of determinations was based on certified standard lyophilized bovine liver—SRM 1577b (National Institute of Standards & Technology, Gaithersburg, MD, USA). The reference material contained zinc, copper and selenium at concentrations of 127 ± 16, 160 ± 8 and 0.73 ± 0.06 (μg/g) respectively. The relative standard deviations obtained in the reference material determinations were 0.3% for zinc, 8.3% for copper and 3.5% for selenium.

### 2.3. Metallothionein-Like Proteins Determinations

The level of metallothionein-like proteins (MTs) were determined using a modified cadmium-hemoglobin affinity assay according to Eaton and Cherian (1991) [24], with the final measurement of cadmium performed by graphite furnace atomic absorption spectrometry with Zeeman correction (Hitachi Z-8270). The detection limit for the cadmium was 0.097 ng/mL. The graph obtained was linear in the concentration range described below, and the equations of the curve were as follows:

Cd (0.5 − 3.0 ng/mL) *y* = 0.093*x* + 0.0208; *R^2^* = 0.9947

### 2.4. Biochemical Parameters

Plasma total antioxidant status (TAS) was measured with the use of a kit test (NX 2332, produced by Randox Laboratories, Antrim, UK). The activity of superoxide dismutase (ESOD) and glutathione peroxide (GPx) in erythrocytes was determined using RANSOD SD125 and RANSEL RS505 tests (Randox Laboratories, Antrim, UK), respectively. The activity of ESOD and GPx were expressed as units per gram of hemoglobin (U/g Hb).

### 2.5. Statistical Analysis

All the results are expressed as means ± SD. STATISTICA 10.0 (StatSoft Inc., Tulsa, OK, USA) was used for all statistical analyses with ANOVA. The significance of the differences for the selected parameters was set using Tukey’s test, after using Bartlett’s test to test for homogeneity of variance. Differences with a *p*-value of less than 0.05 were considered to be statistically significant.

## 3. Results

**Figure 1:**
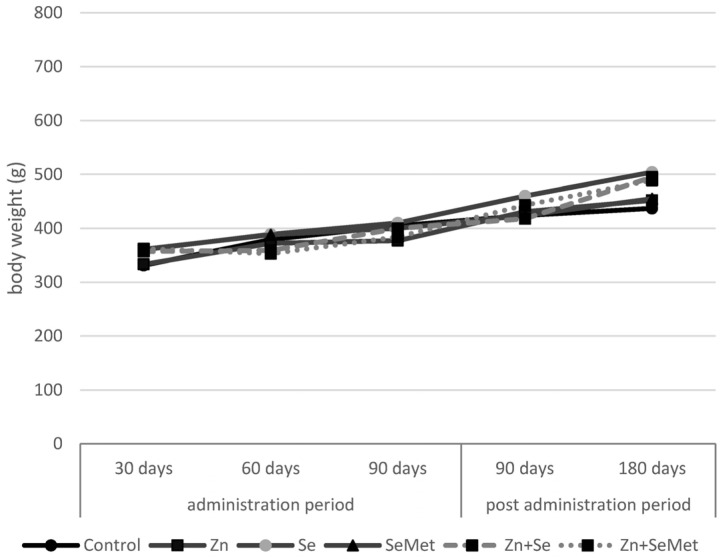
Whole body weight of rats following zinc gluconate (Zn), sodium selenite (Se) and selenomethionine (SeMet) administration given jointly or separately for 30, 60 and 90 days and then after 90 and 180 days post administration.

Figure 1 presents the mean body weights of the rats in the control group and experimental groups during supplementation and after its termination. No statistically significant differences were found between the control and experimental groups. In the rats of experimental groups, no significant differences in the water and feed consumption were observed during the experiment and after its termination, compared to the control group. Similarly, no statistically significant differences between relative and absolute liver and prostate weights were found between individual experimental groups and controls (Table S1).

Table 1 summarizes the results of zinc concentrations in blood, liver and in both parts of the prostate in all experimental groups of rats and in controls. The blood zinc concentrations did not significantly differ in any rats throughout the whole experiment, compared to controls. As shown, liver zinc concentrations were found to be significantly higher in the groups administered only zinc or zinc + selenium (both groups) than in controls as soon as one month after administration, and remained constant until the end of the three-month supplementation period. It should be noted that after the 30-day administration period, zinc concentration was higher (about 26%) in the groups receiving both zinc and selenium than in those receiving only zinc. In the following administration periods, zinc concentration was almost similar in all zinc-administered groups, regardless of whether selenium was given or not. 

The zinc concentration in the prostates of rats receiving zinc alone was significantly higher than that observed in the control group (Table 1). The greatest rise of zinc concentration in the dorso-lateral (DL) lobe of the prostate (over 60% compared to controls) was found after only one month of supplementation. Significantly, this level was maintained not only until the end of the supplementation but also for another three months after its termination (Table 1). In the ventral (V) lobe of the prostate, a significant increase in zinc concentration of almost 50% compared to controls, was observed only after the 90-day supplementation and only in the group receiving zinc alone.

Table 2 shows the data on selenium concentrations in the blood and liver, as well as in both parts of the prostate in the experimental and control groups of rats. Significant elevations of blood selenium concentration compared to controls were noted only in the groups of rats receiving selenium alone (regardless of the form of administration) following 60 and 90 days of administration, while increased liver concentrations were found after as little as 30 days supplementation in all selenium-receiving groups: either administered alone or together with zinc. This significant increase in liver selenium concentration in rats of these groups continued at the same level throughout the supplementation period and remained significantly higher than control values for 90 days after experiment termination.

As in the liver, the selenium level in the DL lobe was significantly higher than control values in all groups of rats receiving selenium (alone or with zinc) throughout the whole administration period (~30%) and for six months after its termination. However, a significant elevation of selenium concentration in the V lobe, following 30, 60 and 90 days of supplementation (~25%, ~50%, ~40% respectively), was only noted in the group of rats receiving SeMet alone or with zinc.

No differences were found between experimental groups and controls with regard to copper concentrations in the blood, liver or either lobe of the prostate, either throughout supplementation or after its termination (Table S2).

**Figure 2:**
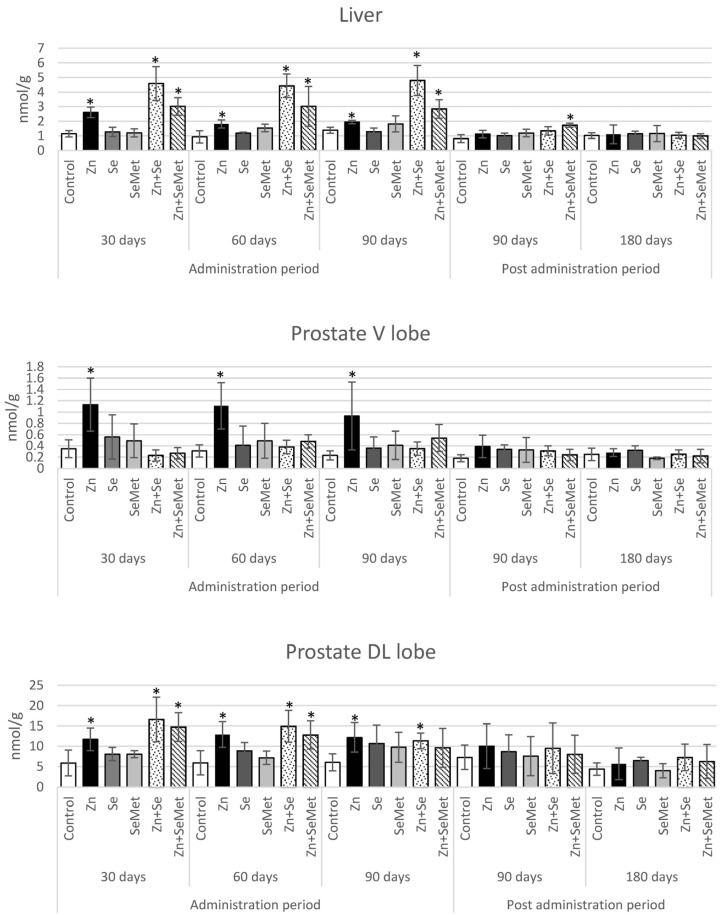
Metallothionein-like proteins level (nmol/g) in rats following zinc gluconate (Zn), sodium selenite (Se) and selenomethionine (SeMet) administration given jointly or separately for 30, 60 and 90 days and then after 90 and 180 days post administration. * Results statistically significant compared to controls, *p* ≤ 0.05.

Figure 2 shows the determinations of the levels of metallothionein-like proteins (MTs) in the liver and in both lobes of the prostate in the experimental and control groups. Compared to controls, the level of MTs in the liver was significantly elevated (2- to almost 5-fold higher) immediately after the 30-day supplementation period in rats given zinc gluconate, either alone or together with selenium (both groups), and what is more, the increase remained at a similar level until the end of supplementation in all groups. The significant increase in MTs level persisted for the longest period in the group of rats receiving zinc together with SeMet: 90 days after termination of supplementation.

Similar patterns of MTs increase were found in the liver and the DL lobe of the prostate. After the first month of zinc + selenium supplementation, a significant increase in MTs levels of almost three-fold was noted, regardless of the form of selenium. After administration of zinc alone, the level of metallothionein-like proteins roughly doubled in the prostate; this is a significant change, but unlike the groups receiving zinc and selenium, MTs induction was also found in both lobes of the prostate (a threefold increase was observed also in the V-lobe). As shown in Figure 2, MTs concentrations in the DL lobe were 10-fold higher than in the V lobe in both the experimental and control groups, which may be due to differences in zinc concentrations in these prostate lobes.

Table 3 presents the selected biochemical parameters used to assess oxidative stress of the rats of experimental and control groups. As shown, no significant differences were revealed in the activity of superoxide dismutase (ESOD), total antioxidant status (TAS) or in the activity of glutathione peroxide (GPx) in the study groups, compared to controls. Therefore, daily supplementation of rats with zinc gluconate, administered separately or jointly with selenium, for up to three months did not induce any change in the oxidation-reduction status.

## 4. Discussion

Zinc and selenium supplementation is recommended in the elderly, especially in men of more advanced age, as their homeostasis is altered during the aging process, due in part to the nutritional deficiencies common in seniors [25,26,27]. A number of studies demonstrated that zinc is particularly critical for the normal functioning of the prostate and an appropriately high level should be maintained in this organ [28,29]. The normal human prostate accumulates the highest levels of zinc of any soft tissue in the body, and zinc is particularly important to its normal functioning, especially regarding the consequences of hormone disturbances [3,30].

Aging is associated with low zinc levels in the prostate and also with prostatic disease, e.g., benign prostatic hyperplasia [31,32,33,34]. Zinc deficiency in the prostate is also one of the major factors implicated in the etiology of prostate cancer [35,36]. It has been shown that zinc deficit has been associated with increased DNA damage in the prostate during oxidative stress [34]. Specifically, Zn deficient prostate cells have greater DNA damage and altered expression of genes associated with this damage, indicating that marginal Zn intake may sensitize the prostate to oxidative damage [34].

Some studies indicate that selenium deficiency may also play a significant role in the etiology of prostate cancer [37]. However, the function of selenium in the prostate has not as yet been specified. Epidemiological studies have suggested an inverse association between blood selenium and incidence of prostate cancer [38,39]. Dennert et al. (2011) observed that the presence of high levels of selenium in the diet decreases the incidence of a number of cancers, including prostate cancer [40].

While a literature review shows that no studies aimed at assessing the effect of selenium + zinc supplementation on the bioavailability of these two elements in the prostate have been carried out, this particular supplementation model is frequently applied in the prevention of prostate diseases due to the fact that pharmaceutical preparations comprise both elements [41]. Generally, little is known of zinc and selenium interactions in nutrition. However, cellular studies demonstrate that they are significant, and that selenium compounds may express their antioxidant or oxidant potential through differences in the levels of released zinc [42]. Selenium-generated perturbation of zinc homeostasis was suggested as a mechanism responsible for the stimulation of metallothionein synthesis [43]. Metallothioneins are low-molecular-weight, sulfhydryl-rich proteins that are known to bind zinc predominantly. The binding of seemingly redox inactive zinc ions allows metallothionein to play a central role in oxidoreductive cellular metabolism, cellular zinc distribution and homeostasis [44].

A review of recent findings by Maret (2000) confirmed selenium compounds as oxidants of metallothionein in vivo, which may act to release zinc from metallothionein, and possibly stimulate thionein synthesis via the zinc-mediated mechanism [22]. The mechanism of the reaction was suggested to proceed through an activated selenenyl sulphide (R-Se-S-G) intermediate which, in turn, oxidizes the zinc-thiolate cluster of metallothionein to form R-Se-S-MT with the concomitant release of zinc during oxidation. Selenium compounds also catalyze the release of zinc from metallothionein in peroxidation and thiol/disulfide-interchange reactions [45]. Hence, one of the aims of our study was to examine the potential interactions between zinc and selenium administered jointly or separately on induction of MTs in the liver and prostate, and thus on the bioavailability these two elements for the prostate, especially the ventral lobe, because zinc levels in this lobe decrease with age [46]. Unlike in the ventral lobe, the zinc content in the dorso-lateral lobe did not differ between young and old rats [46]. It should be underlined that the peripheral lobe of the human prostate accumulates the greatest amount of zinc, reaching a level five- to ten-fold higher than other prostate lobes [28] and in this sense it is considered to be comparable with the dorsolateral lobe of the rat prostate, in which zinc levels are also almost ten-fold higher than in the ventral lobe [47]. Only in the glandular epithelium of the DL lobe, comprising highly specialized zinc-accumulating cells of the prostate, is such a high level of zinc essential; it inhibits m-aconitase, spares citrate oxidation in the Krebs cycle, and provides large amounts of citrate for secretion into prostatic fluid. In contrast, the glandular cells of the ventral lobe do not accumulate zinc and are citrate-oxidizing cells typical of most mammalian cells [5]. The specific role of selenium in the prostate is not known. It is believed that this element functions as an antioxidant in the enzyme selenium-gluthatione-peroxidase. Moreover selenium has been shown to improve the production of sperm and sperm motility. In contrast to zinc, the selenium levels of the ventral prostate are around twice those of the dorso-lateral lobe. This may be associated with prostate epithelial selenoproteins (15 kD) which are found in the epithelial cells of the ventral prostate [48]. Based on the potential biochemical implications of zinc + selenium application on metallothionein/thionein status discussed above, our results seem to confirm that the elevated metallothionein levels induced by zinc can be expected in different organs following zinc + selenium supplementation. Liver MTs concentration was almost two–fold higher in rats receiving zinc with selenium than in those receiving zinc alone, and no significant changes in MTs level were observed until the end of their administration.

The liver of rats receiving zinc + selenium demonstrated both a significant increase in zinc level and greater MT concentration. In these two groups (Zn + Se and Zn + SeMet) the increase in zinc concentration was found after one month of supplementation, compared to controls. These results indicate that selenium administered jointly with zinc induced a significant increase in liver zinc concentration, most likely due to secondary MTs induction by zinc released from MTs by interaction with selenium, which is in accordance with Maret (2000), as noted earlier [22]. A similar zinc-selenium interaction demonstrated by an elevated zinc concentration was earlier evidenced by Chmielnicka et al. (1988) in rats given both elements in a single dose [49]. The study also demonstrated that selenium increased zinc concentration in the liver and other organs, but the prostate was not investigated [49].

A similar dependence was observed in the dorso-lateral lobe, although the difference in elevated level of MTs between groups was not as obvious as in the liver. Interestingly, the group of rats administered with zinc alone was the only group to demonstrate a significant increase in zinc concentration in the DL lobe of the prostate that continued throughout the supplementation period and three months after its termination. Although increased zinc levels were also observed in this part of the prostate during the supplementation period, they were not statistically significant in rats administrated with zinc + selenium.

Quite a different effect was observed in the ventral lobe, where elevated MTs levels only remained stable throughout the whole administration period in the zinc group. Zinc levels were significantly higher than in the control group after only three months, which might have resulted from the physiological decline of zinc concentrations with age. However, zinc levels in this part of the prostate were unchanged throughout the whole three-month supplementation period. This indicates that the administration of zinc alone should be considered to achieve the best effects of supplementation for increasing zinc levels in both lobes of the prostate (notably in the V lobe).

In the case of selenium, the highest levels were evidenced in both parts of the prostate only after application of SeMet, regardless of the mode of zinc administration. SeMet has been found to be more effective in increasing apparent selenium status because it is non-specifically incorporated into proteins, such as hemoglobin or albumin, in place of methionine [50].

The level of copper was also assessed in all rats subjected to supplementation, as it could interact with zinc during long-term zinc supplementation. The pool of zinc and copper is always in accordance with the balance between zinc-metallothioneins and copper-metallothioneins. Despite finding an increase in the concentrations of both MTs and zinc in the livers and prostates of the rats under study, no changes in copper levels were observed in the blood or the investigated organs.

## 5. Conclusions

To sum up, our study is the first to demonstrate the effect of selenium on the bioavailability of zinc in the prostate following administration of different forms of zinc and selenium supplementation. The highest zinc bioavailability and highest consequent prostate concentrations were found after supplementation with zinc alone. The co-administration of zinc and selenium does not significantly affect the bioavailability of selenium in the prostate. The results of our studies indicate that these elements should not be administered jointly in supplementation. These findings, after confirmation, should be an important indication for physicians regarding the administration of dietary supplements.

## Figures and Tables

**Table 1:** Zinc level in the blood, liver and prostate of rats following zinc gluconate (Zn), sodium selenite (Se) and selenomethionine (SeMet) administration given jointly or separately for 30, 60 and 90 days and then after 90 and 180 days post administration.

	Blood (μg/mL)	Liver (μg/g w.t.)	Prostate (μg/g w.t.)	
			V Lobe	DL Lobe
**Administration Period**				
**30-day**				
Control	5.67 ± 0.18	38.77 ± 1.73	28.03 ± 7.21	240.27 ± 66.58
Zn	5.83 ± 0.21	42.80 ± 1.82 *	31.89 ± 3.14	396.77 ± 63.18 *
Se	5.63 ± 0.14	41.69 ± 1.73	21.01 ± 6.45	298.33 ± 22.99
SeMet	5.97 ± 0.22	41.07 ± 2.39	20.98 ± 5.55	238.22 ± 42.61
Zn + Se	5.98 ± 0.20	52.89 ± 2.57 *	21.54 ± 6.21	338.97 ± 87.62
Zn + SeMet	5.67 ± 0.31	50.53 ± 5.40 *	21.54 ± 4.98	309.13 ± 82.32
**60-day**				
Control	5.83 ± 0.34	37.23 ± 2.84	25.63 ± 4.21	256.33 ± 40.15
Zn	5.43 ± 0.21	43.70 ± 3.78 *	29.56 ± 3.11	382.21 ± 61.22 *
Se	5.83 ± 0.14	38.95 ± 4.64	24.39 ± 2.66	284.33 ± 39.21
SeMet	5.37 ± 0.25	38.02 ± 4.60	20.48 ± 3.74	231.22 ± 48.71
Zn + Se	5.98 ± 0.33	44.72 ± 4.01 *	20.14 ± 4.15	341.11 ± 70.55
Zn + SeMet	5.77 ± 0.24	44.90 ± 2.36 *	22.65 ± 3.11	298.77 ± 41.20
**90-day**				
Control	5.43 ± 0.24	34.94 ± 2.68	20.34 ± 2.83	244.81 ± 28.11
Zn	5.43 ± 0.19	43.50 ± 3.77 *	29.39 ± 5.38 *	374.86 ± 38.35 *
Se	5.16 ± 0.27	37.04 ± 1.56	23.67 ± 6.31	285.77 ± 49.42
SeMet	5.83 ± 0.24	40.67 ± 3.44	19.74 ± 4.57	225.20 ± 68.75
Zn + Se	5.64 ± 0.18	44.13 ± 4.10 *	16.83 ± 7.28	306.08 ± 49.00
Zn + SeMet	5.76 ± 0.36	43.52 ± 3.17 *	21.73 ± 11.23	301.98 ± 46.35
**Post Administration Period**				
**90-day**				
Control	5.25 ± 0.31	35.42 ± 2.68	15.25 ± 4.10	275.23 ± 44.29
Zn	5.87 ± 0.36	41.29 ± 4.19	10.53 ± 6.32	375.18 ± 64.09 *
Se	5.16 ± 0.22	37.19 ± 3.71	13.75 ± 6.02	232.16 ± 26.50
SeMet	5.76 ± 0.38	35.48 ± 4.14	14.69 ± 4.32	263.68 ± 51.97
Zn + Se	5.44 ± 0.26	38.08 ± 3.16	10.32 ± 3.06	267.30 ± 75.21
Zn + SeMet	5.91 ± 0.41	38.91 ± 2.83	13.25 ± 3.37	275.65 ± 37.11
**180-day**				
Control	5.33 ± 0.35	36.41 ± 3.85	14.81 ± 5.54	261.56 ± 69.86
Zn	5.04 ± 0.28	40.08 ± 1.81	11.89 ± 1.88	303.30 ± 91.57
Se	4.98 ± 0.24	35.95 ± 2.53	12.36 ± 4.07	282.10 ± 8.64
SeMet	5.49 ± 0.28	39.56 ± 5.57	11.91 ± 3.24	193.87 ± 74.09
Zn + Se	4.87 ± 0.31	35.96 ± 1.78	13.38 ± 7.02	258.20 ± 64.57
Zn + SeMet	5.88 ± 0.42	38.13 ± 1.88	10.52 ± 2.94	237.64 ± 50.23

All values are expressed as means ± SD; * Results statistically significant compared to controls, *p* ≤ 0.05 V lobe: ventral lobe; DL lobe: dorso-lateral lobe.

**Table 2:** Selenium level in the blood, liver and prostate of rats following zinc gluconate (Zn), sodium selenite (Se) and selenomethionine (SeMet) administration given jointly or separately for 30, 60 and 90 days and then after 90 and 180 days post administration.

	Blood (μg/mL)	Liver (μg/g w.t.)	Prostate (μg/g w.t.)	
			V Lobe	DL Lobe
**Administration Period**				
**30-day**				
Control	0.51 ± 0.03	1.18 ± 0.02	0.41 ± 0.05	0.23 ± 0.03
Zn	0.53 ± 0.02	1.17 ± 0.07	0.37 ± 0.05	0.28 ± 0.06
Se	0.56 ± 0.03	1.24 ± 0.02 *	0.40 ± 0.08	0.30 ± 0.03 *
SeMet	0.56 ± 0.03	1.27 ± 0.03 *	0.52 ± 0.05 *	0.31 ± 0.02 *
Zn + Se	0.52 ± 0.04	1.24 ± 0.05 *	0.41 ± 0.04	0.32 ± 0.02 *
Zn + SeMet	0.50 ± 0.02	1.26 ± 0.05 *	0.51 ± 0.05 *	0.31 ± 0.02 *
**60-day**				
Control	0.53 ± 0.02	1.22 ± 0.02	0.41 ± 0.07	0.24 ± 0.04
Zn	0.51 ± 0.02	1.28 ± 0.09	0.42 ± 0.04	0.27 ± 0.02
Se	0.58 ± 0.02 *	1.41 ± 0.09 *	0.48 ± 0.06	0.36 ± 0.06 *
SeMet	0.56 ± 0.01 *	1.40 ± 0.12 *	0.63 ± 0.08 *	0.38 ± 0.04 *
Zn + Se	0.55 ± 0.02	1.31 ± 0.06 *	0.50 ± 0.06	0.34 ± 0.05 *
Zn + SeMet	0.52 ± 0.03	1.31 ± 0.06 *	0.62 ± 0.07 *	0.36 ± 0.04 *
**90-day**				
Control	0.51 ± 0.03	1.21 ± 0.08	0.45 ± 0.07	0.28 ± 0.02
Zn	0.52 ± 0.02	1.27 ± 0.09	0.49 ± 0.05	0.27 ± 0.03
Se	0.59 ± 0.03 *	1.41 ± 0.04 *	0.49 ± 0.04	0.42 ± 0.04 *
SeMet	0.58 ± 0.03 *	1.39 ± 0.04 *	0.69 ± 0.09 *	0.39 ± 0.02 *
Zn + Se	0.51 ± 0.04	1.36 ± 0.05 *	0.52 ± 0.04	0.37 ± 0.07 *
Zn + SeMet	0.50 ± 0.04	1.41 ± 0.06 *	0.62 ± 0.05 *	0.39 ± 0.06 *
**Post Administration Period**				
**90-day**				
Control	0.51 ± 0.03	1.13 ± 0.10	0.36 ± 0.04	0.26 ± 0.03
Zn	0.52 ± 0.02	1.20 ± 0.07	0.36 ± 0.07	0.27 ± 0.02
Se	0.52 ± 0.02	1.24 ± 0.02 *	0.30 ± 0.03	0.32 ± 0.02 *
SeMet	0.56 ± 0.03	1.37 ± 0.12 *	0.39 ± 0.03	0.31 ± 0.01 *
Zn + Se	0.50 ± 0.02	1.24 ± 0.08 *	0.44 ± 0.05	0.33 ± 0.04 *
Zn + SeMet	0.55 ± 0.04	1.24 ± 0.07 *	0.42 ± 0.07	0.37 ± 0.05 *
**180-day**				
Control	0.51 ± 0.04	1.09 ± 0.09	0.30 ± 0.02	0.26 ± 0.02
Zn	0.51 ± 0.02	1.18 ± 0.05	0.32 ± 0.05	0.30 ± 0.03
Se	0.50 ± 0.02	1.26 ± 0.08	0.38 ± 0.08	0.33 ± 0.04 *
SeMet	0.54 ± 0.05	1.21 ± 0.05	0.32 ± 0.03	0.30 ± 0.01 *
Zn + Se	0.50 ± 0.03	1.09 ± 0.08	0.31 ± 0.08	0.31 ± 0.03 *
Zn + SeMet	0.55 ± 0.05	1.27 ± 0.13	0.31 ± 0.07	0.36 ± 0.04 *

All values are expressed as means ± SD; * Results statistically significant compared to controls, *p* ≤ 0.05.

**Table 3:** Selected biochemical parameters in rats following zinc gluconate (Zn), sodium selenite (Se) and selenomethionine (SeMet) administration given jointly or separately for 30, 60 and 90 days and then after 90 and 180 days post-administration.

	TAS (mM/L Plasma)	ESOD (U/g Hb)	GPx (U/g Hb)
**Administration Period**			
**30-day**			
Control	1.07 ± 0.13	3282.5 ± 423.6	21.85 ± 1.34
Zn	1.13 ± 0.14	3690.0 ± 440.3	21.79 ± 2.37
Se	1.20 ± 0.16	3614.0 ± 164.8	24.18 ± 2.33
SeMet	1.06 ± 0.20	3384.0 ± 262.3	22.03 ± 1.12
Zn + Se	1.16 ± 0.16	3614.0 ± 589.2	22.61 ± 1.39
Zn + SeMet	1.16 ± 0.15	3512.0 ± 234.0	21.89 ± 1.54
**60-day**			
Control	1.06 ± 0.09	3393.4 ± 388.8	21.89 ± 1.88
Zn	1.06 ± 0.10	3802.5 ± 393.3	21.98 ± 2.21
Se	1.01 ± 0.06	3420.0 ± 191.1	24.81 ± 1.94
SeMet	1.02 ± 0.07	3280.0 ± 362.2	23.05 ± 2.59
Zn + Se	0.99 ± 0.11	3724.1 ± 423.1	23.22 ± 1.99
Zn + SeMet	0.96 ± 0.09	3632.2 ± 352.3	22.33 ± 2.19
**90-day**			
Control	0.99 ± 0.03	3803.3 ± 192.9	21.64 ± 1.70
Zn	0.95 ± 0.11	4220.0 ± 624.1	22.94 ± 3.00
Se	0.90 ± 0.10	3470.0 ± 435.6	25.35 ± 4.53
SeMet	0.99 ± 0.12	3520.0 ± 449.2	22.65 ± 2.89
Zn + Se	0.91 ± 0.07	3970.0 ± 701.2	23.11 ± 2.96
Zn + SeMet	0.94 ± 0.05	3828.0 ± 877.7	21.83 ± 1.31
**Post Administration Period**			
**90-day**			
Control	0.76 ± 0.03	3230.0 ± 372.4	20.62 ± 1.58
Zn	076 ± 0.08	3542.5 ± 352.8	21.75 ± 1.51
Se	0.76 ± 0.09	3438.0 ± 222.1	24.31 ± 2.49
SeMet	0.83 ± 0.05	3102.0 ± 368.8	21.32 ± 1.56
Zn + Se	0.77 ± 0.10	3530.0 ± 593.5	21.38 ± 1.47
Zn + SeMet	0.73 ± 0.15	3556.0 ± 421.0	20.83 ± 1.70
**180-day**			
Control	0.74 ± 0.07	3443.1 ± 325.6	20.68 ± 2.09
Zn	0.91 ± 0.11	3802.0 ± 419.7	20.02 ± 1.33
Se	0.89 ± 0.09	3490.0 ± 310.4	22.37 ± 1.78
SeMet	0.89 ± 0.10	3676.2 ± 290.0	20.68 ± 1.03
Zn + Se	0.91 ± 0.10	3381.0 ± 361.9	21.93 ± 1.57
Zn + SeMet	0.89 ± 0.16	3880.2 ± 411.1	20.39 ± 1.79

All values are expressed as means ± SD.

## Supplementary Materials

The following are available online at http://www.mdpi.com/2072-6643/8/10/601/s1, Table S1: Liver and prostate weight (g) (relative weight (g/100 g b.w.)) in rats following zinc gluconate (Zn), sodium selenite (Se) and selenomethionine (SeMet) administration given jointly or separately for 30, 60 and 90 days and then after 90 and 180 days post administration. Table S2: Copper level in the blood, liver and prostate of rats following zinc gluconate (Zn), sodium selenite (Se) and selenomethionine (SeMet) administration given jointly or separately for 30, 60 and 90 days and then after 90 and 180 days post administration.
