# Supplementary material for: The Effect of Zinc and Selenium Supplementation Mode on Their Bioavailability in the Rat Prostate. Should Administration Be Joint or Separate?
Source: Nutrients. 2016 Oct 4;8(10):601. doi: 10.3390/nu8100601 (PMC5083989; doi:10.3390/nu8100601)
Supplement: Supplementary file 1 [file nutrients-08-00601-s001.pdf]

Supplementary Materials: The Effect of Mode of Zinc and Selenium Supplementation on Their Bioavailability in the Rat Prostate. Should Administration Be Joint or Separate?

Adam Daragó, Andrzej Sapota, Marzenna Nasiadek, Michał Klimczak and Anna Kilanowicz

Table S1. Liver and prostate weight (g) (relative weight (g/100 g b.w.)) in rats following zinc gluconate (Zn), sodium selenite (Se) and selenomethionine (SeMet) administration given jointly or separately for 30, 60 and 90 days and then after 90 and 180 days post administration.

	Liver	Prostate	
		V Lobe	DL Lobe
**Administration Period**			
30-day			
Control	9.47 ± 0.81 (2.86 ± 0.38)	0.43 ± 0.04 (0.13 ± 0.01)	0.46 ± 0.05 (0.14 ± 0.02)
Zn	9.52 ± 0.95 (2.86 ± 0.40)	0.37 ± 0.03 (0.11 ± 0.01)	0.37 ± 0.08 (0.11 ± 0.03)
Se	9.98 ± 0.34 (2.70 ± 0.21)	0.41 ± 0.03 (0.11 ± 0.02)	0.37 ± 0.05 (0.10 ± 0.02)
SeMet	10.18 ± 0.62 (2.82 ± 0.29)	0.40 ± 0.04 (0.11 ± 0.02)	0.36 ± 0.06 (0.10 ± 0.02)
Zn + Se	9.56 ± 0.28 (2.68 ± 0.11)	0.39 ± 0.03 (0.11 ± 0.02)	0.36 ± 0.06 (0.10 ± 0.02)
Zn + SeMet	9.92 ± 0.42 (2.74 ± 0.24)	0.40 ± 0.07 (0.11 ± 0.02)	0.36 ± 0.0.9 (0.10 ± 0.03)
60-day			
Control	10.60 ± 0.91 (2.85 ± 0.16)	0.41 ± 0.04 (0.11 ± 0.02)	0.48 ± 0.08 (0.13 ± 0.03)
Zn	9.49 ± 0.61 (2.55 ± 0.16)	0.35 ± 0.02 (0.09 ± 0.01)	0.37 ± 0.04(0.10 ± 0.02)
Se	10.34 ± 0.71 (2.66 ± 0.12)	0.37 ± 0.06 (0.09 ± 0.02)	0.43 ± 0.06 (0.11 ± 0.02)
SeMet	10.63 ± 0.81 (2.74 ± 0.22)	0.39 ± 0.02 (0.10 ± 0.01)	0.43 ± 0.05 (0.11 ± 0.02)
Zn + Se	9.26 ± 0.72 (2.58 ± 0.18)	0.39 ± 0.02 (0.11 ± 0.01)	0.36 ± 0.09 (0.08 ± 0.03)
Zn + SeMet	8.85 ± 0.97 (2.51 ± 0.12)	0.38 ± 0.03 (0.09 ± 0.01)	0.38 ± 0.03 (0.10 ± 0.01)
90-day			
Control	11.09 ± 0.74 (2.85 ± 0.20)	0.46 ± 0.04 (0.11 ± 0.01)	0.47 ± 0.08 (0.11 ± 0.02)
Zn	10.13 ± 0.63 (2.69 ± 0.15)	0.35 ± 0.08 (0.09 ± 0.02)	0.37 ± 0.09 (0.09 ± 0.02)
Se	10.75 ± 0.89 (2.62 ± 0.16)	0.45 ± 0.06 (0.11 ± 0.02)	0.37 ± 0.12(0.09 ± 0.03)
SeMet	11.05 ± 0.61 (2.76 ± 0.13)	0.40 ± 0.02 (0.10 ± 0.01)	0.37 ± 0.09 (0.08 ± 0.02)
Zn + Se	10.48 ± 0.36 (2.62 ± 0.09)	0.44 ± 0.04 (0.11 ± 0.01)	0.40 ± 0.07 (0.10 ± 0.02)
Zn + SeMet	10.13 ± 0.42 (2.64 ± 0.14)	0.42 ± 0.03 (0.11 ± 0.01)	0.42 ± 0.03 (0.11 ± 0.01)
**Post Administration Period**			
90-day			
Control	12.23 ± 0.73 (3.02 ± 0.17)	0.45 ± 0.02 (0.11 ± 0.02)	0.45 ± 0.02 (0.11 ± 0.02)
Zn	12.02 ± 0.58 (2.79 ± 0.15)	0.43 ± 0.19 (0.10 ± 0.04)	0.47 ± 0.19 (0.11 ± 0.04)
Se	12.28 ± 0.88 (2.67 ± 0.19)	0.37 ± 0.07 (0.08 ± 0.02)	0.46 ± 0.07 (0.10 ± 0.02)
SeMet	12.90 ± 0.81 (3.04 ± 0.26)	0.42 ± 0.15 (0.10 ± 0.03)	0.42 ± 0.15 (0.10 ± 0.03)
Zn + Se	11.70 ± 0.92 (2.80 ± 0.25)	0.42 ± 0.10 (0.10 ± 0.02)	0.42 ± 0.10 (0.10 ± 0.02)
Zn + SeMet	11.77 ± 0.87 (2.66 ± 0.28)	0.44 ± 0.01 (0.10 ± 0.01)	0.44 ± 0.13 (0.10 ± 0.03)
180-day			
Control	12.58 ± 0.67 (2.88 ± 0.15)	0.57 ± 0.06 (0.13 ± 0.02)	0.48 ± 0.10 (0.11 ± 0.03)
Zn	12.24 ± 0.71 (2.71 ± 0.17)	0.45 ± 0.08 (0.10 ± 0.02)	0.45 ± 0.12 (0.10 ± 0.03)
Se	13.45 ± 0.81 (2.67 ± 0.19)	0.45 ± 0.09 (0.09 ± 0.02)	0.40 ± 0.09 (0.08 ± 0.02)
SeMet	12.39 ± 0.87 (2.72 ± 0.29)	0.50 ± 0.09 (0.11 ± 0.02)	0.36 ± 0.09 (0.08 ± 0.02)
Zn + Se	13.36 ± 0.52 (2.70 ± 0.15)	0.59 ± 0.09 (0.12 ± 0.02)	0.54 ± 0.09 (0.11 ± 0.02)
Zn + SeMet	13.79 ± 0.74 (2.82 ± 0.23)	0.49 ± 0.11 (0.10 ± 0.02)	0.39 ± 0.11 (0.08 ± 0.02)

All values are expressed as means ± SD; * Results statistically significant compared to controls, $p \leq 0.05$.

Table S2. Copper level in the blood, liver and prostate of rats following zinc gluconate (Zn), sodium selenite (Se) and selenomethionine (SeMet) administration given jointly or separately for 30, 60 and 90 days and then after 90 and 180 days post administration

	Blood (µg/mL)	Liver (µg/g w.t.)	Prostate (µg/g w.t.)	
			V lobe	DL lobe
**Administration Period**				
30-day				
Control	1.02 ± 0.07	4.37 ± 0.29	2.03 ± 0.37	3.21 ± 0.48
Zn	1.03 ± 0.08	3.92 ± 0.50	1.98 ± 0.24	3.41 ± 0.32
Se	1.00 ± 0.07	4.19 ± 0.17	2.14 ± 0.34	2.95 ± 0.24
SeMet	1.01 ± 0.04	3.83 ± 0.33	2.36 ± 0.29	3.15 ± 0.26
Zn + Se	1.01 ± 0.04	4.23 ± 0.37	2.34 ± 0.41	3.16 ± 0.24
Zn + SeMet	1.02 ± 0.07	4.07 ± 0.57	2.28 ± 0.41	3.31 ± 0.39
60-day				
Control	1.04 ± 0.08	5.21 ± 0.63	2.27 ± 0.41	3.12 ± 0.42
Zn	1.05 ± 0.07	5.22 ± 0.48	2.21 ± 0.56	3.42 ± 0.56
Se	0.98 ± 0.04	4.39 ± 0.53	2.08 ± 0.29	3.31 ± 0.47
SeMet	1.08 ± 0.08	4.72 ± 0.63	2.45 ± 0.35	3.16 ± 0.34
Zn + Se	1.01 ± 0.05	5.36 ± 0.70	2.31 ± 0.51	3.37 ± 0.48
Zn + SeMet	1.05 ± 0.04	4.47 ± 0.43	2.45 ± 0.34	3.42 ± 0.49
90-day				
Control	1.03 ± 0.05	3.79 ± 0.28	2.24 ± 0.37	2.98 ± 0.39
Zn	0.99 ± 0.04	3.72 ± 0.62	2.04 ± 0.27	3.35 ± 0.62
Se	0.99 ± 0.05	3.56 ± 0.24	2.38 ± 0.38	3.15 ± 0.38
SeMet	1.01 ± 0.05	3.58 ± 0.40	2.40 ± 0.40	3.24 ± 0.41
Zn + Se	1.08 ± 0.08	3.66 ± 0.41	2.34 ± 0.43	3.20 ± 0.41
Zn + SeMet	1.09 ± 0.08	3.56 ± 0.45	2.47 ± 0.51	3.41 ± 0.53
**Post Administration Period**				
90-day				
Control	1.07 ± 0.08	3.59 ± 0.35	2.18 ± 0.37	3.06 ± 0.41
Zn	0.99 ± 0.05	4.02 ± 0.15	2.05 ± 0.28	2.93 ± 0.37
Se	0.99 ± 0.06	3.79 ± 0.51	2.43 ± 0.29	3.09 ± 0.43
SeMet	1.07 ± 0.05	3.91 ± 0.39	2.01 ± 0.39	3.34 ± 0.55
Zn + Se	0.98 ± 0.04	3.71 ± 0.16	2.08 ± 0.39	3.13 ± 0.36
Zn + SeMet	1.00 ± 0.04	3.66 ± 0.24	2.35 ± 0.24	3.38 ± 0.49
180-day				
Control	1.07 ± 0.08	4.37 ± ± 0.63	1.89 ± 0.42	2.94 ± 0.39
Zn	1.01 ± 0.02	4.97 ± 0.78	2.08 ± 0.41	3.02 ± 0.42
Se	0.98 ± 0.04	5.31 ± 1.28	2.24 ± 0.39	3.09 ± 0.39
SeMet	1.05 ± 0.04	5.8 ± 1.05	2.19 ± 0.41	3.13 ± 0.45
Zn + Se	1.01 ± 0.02	4.65 ± 1.07	2.13 ± 0.27	2.88 ± 0.32
Zn + SeMet	1.01 ± 0.03	5.03 ± 0.39	1.96 ± 0.39	3.21 ± 0.46

All values are expressed as means ± SD; * Results statistically significant compared to controls, $p \leq 0.05$.
